# Metagenome Assembly and Metagenome-Assembled Genome of “*Candidatus* Aramenus sulfurataquae” from Thermal Sediments from the Los Azufres Volcanic Complex

**DOI:** 10.1128/MRA.00379-21

**Published:** 2021-09-30

**Authors:** Roberto Marín-Paredes, Yunuen Tapia-Torres, Esperanza Martínez-Romero, Mauricio Quesada, Luis E. Servín-Garcidueñas

**Affiliations:** a Laboratorio de Microbiómica, Escuela Nacional de Estudios Superiores Unidad Morelia, Universidad Nacional Autónoma de México, Morelia, Michoacán, México; b Laboratorio Nacional de Análisis y Síntesis Ecológica, Escuela Nacional de Estudios Superiores Unidad Morelia, Morelia, Michoacán, México; c Centro de Ciencias Genómicas, Universidad Nacional Autónoma de México, Cuernavaca, Morelos, México; d Instituto de Investigaciones en Ecosistemas y Sustentabilidad, Universidad Nacional Autónoma de México, Morelia, Michoacán, México; Indiana University, Bloomington

## Abstract

A plethora of hot springs are found at the Los Azufres volcanic complex in Mexico, and studies are needed to determine their microbial genomic diversity. Here, we report a metagenome of hot spring sediments and a metagenome-assembled genome of “*Candidatus* Aramenus sulfurataquae.” This study reveals novel genomic sequences of *Sulfolobales* archaea.

## ANNOUNCEMENT

Archaea from the order *Sulfolobales* are commonly found in acidic high-temperature environments (1, 2). A large diversity of environmental *Sulfolobales* strains remains to be described. We are interested in characterizing the genomic diversity of *Sulfolobales* strains inhabiting extreme environments found in the Los Azufres volcanic area in western Mexico. Previously, we obtained a genome assembly for the “*Candidatus* Aramenus” genus from a hot spring metagenome from water samples (65°C, pH 3.6) (3). The phylogenetic relationship of the *Aramenus* genus within *Sulfolobales* has been investigated recently (4). Interestingly, current diversity studies have detected *Aramenus* archaea at the El Chichón volcano in Chiapas, Mexico (5). Additional genomes are required to better understand the phylogenetic position, genomic diversity, and evolutionary history of the *Aramenus* genus. We describe a new metagenome-assembled genome (MAG) for “*Candidatus* Aramenus sulfurataquae” strain AZ2 from thermal sediments within Los Azufres.

A sediment sample was collected from a hot spring (88.5°C, pH 2.8) at Los Azufres in May 2016. DNA was isolated using the DNeasy UltraClean microbial kit (Qiagen GmbH, Germany). Library preparation was performed using a DNA preparation kit (Illumina Inc., San Diego, CA, USA) according to the manufacturer's instructions. Sequencing was performed at the Laboratorio Nacional de Análisis y Síntesis Ecológica (Morelia, Mexico) on an Illumina NextSeq550 platform, producing 2 × 75-bp paired-end reads. Reads were assessed using FastQC v.0.11.8 (with default settings) (6) and were filtered for quality (scores of ≥Q30) and adaptor sequences using Trim Galore v.0.6.6 (7). Reads were assembled *de novo* with SPAdes v.3.8.2 (8, 9). Metagenomic annotation was performed using the Integrated Microbial Genome and Microbiomes (IMG/M) pipeline (10). The MAG of “*Candidatus* Aramenus sulfurataquae” was recovered by mapping the reads against the genome of “*Candidatus* Aramenus sulfurataquae” strain AZ1 (GenBank accession number ASRH00000000.1) using bowtie2 v.2.3.4.3 (11) with the -very-sensitive mode. Mapped reads were assembled with SPAdes v.3.12.0 (8, 9) using *k*-mer values of 21, 33, 43, 53, and 63. MAG quality was revised with CheckM v.1.0.13 (with default settings) (12–15). MAG annotation was performed using the NCBI Prokaryotic Genome Annotation Pipeline (PGAP) v.5.2 (16). Average nucleotide identity (ANI) values were calculated as proposed previously (17), using the ANI calculator from the Konstantinidis laboratory (http://enve-omics.ce.gatech.edu/ani) (18).

Metagenomic reads (60,553,942 reads) were assembled into 1,718 contigs (*N*_50_, 7,585 bp) with a minimum length of 1,000 bp, containing 11,279,257 bp. The metagenome revealed *Sulfolobales* sequences related to the *Acidianus*, *Sulfolobus*, and *Aramenus* genera. A 1,681,874-bp draft MAG comprising 379 contigs (*N*_50_, 11,611 bp), with a GC content of 47.8% and genome coverage of 84.31×, could be obtained from the metagenome. The MAG was estimated to be 99.29% complete, with no detected contamination. MAG annotation predicted 2,103 genes and revealed a 1,498-bp 16S rRNA gene that shares 100% sequence identity with the gene from “*Candidatus* Aramenus sulfurataquae” strain AZ1. Genes involved in carbon fixation and sulfur metabolism could be detected. The MAG exhibited an ANI value of 97.91% against the “*Candidatus* Aramenus sulfurataquae” strain AZ1 genome, suggesting that they correspond to the same species. We identified a “*Candidatus* Aramenus sulfurataquae” population in a new niche within Los Azufres with higher temperature and lower pH.

### Data availability.

Raw sequencing data are available in the NCBI Sequence Read Archive (SRA) under accession number SRR7716004. The metagenome assembly is available in GenBank under accession number JAFNEM000000000. The metagenome functional annotation is available from the JGI Genome Portal under accession number 3300029921. The annotated MAG for “*Candidatus* Aramenus sulfurataquae” strain AZ2 is available in GenBank under accession number JAFLRL000000000.
